# Antiarrhythmic Effects of Dantrolene in Patients with Catecholaminergic Polymorphic Ventricular Tachycardia and Replication of the Responses Using iPSC Models

**DOI:** 10.1371/journal.pone.0125366

**Published:** 2015-05-08

**Authors:** Kirsi Penttinen, Heikki Swan, Sari Vanninen, Jere Paavola, Annukka M. Lahtinen, Kimmo Kontula, Katriina Aalto-Setälä

**Affiliations:** 1 BioMediTech, University of Tampere, Tampere, Finland; 2 School of Medicine, University of Tampere, Tampere, Finland; 3 Heart and Lung Center, Helsinki University Hospital, Helsinki, Finland; 4 Heart Hospital, Tampere University Hospital, Tampere, Finland; 5 Minerva Foundation Institute for Medical Research, Helsinki, Finland; 6 Department of Medicine, University of Helsinki and Helsinki University Hospital, Helsinki, Finland; Johns Hopkins University SOM, UNITED STATES

## Abstract

Catecholaminergic polymorphic ventricular tachycardia (CPVT) is a highly malignant inherited arrhythmogenic disorder. Type 1 CPVT (CPVT1) is caused by cardiac ryanodine receptor (*RyR2*) gene mutations resulting in abnormal calcium release from sarcoplasmic reticulum. Dantrolene, an inhibitor of sarcoplasmic Ca^2+^ release, has been shown to rescue this abnormal Ca^2+^ release in vitro. We assessed the antiarrhythmic efficacy of dantrolene in six patients carrying various *RyR2* mutations causing CPVT. The patients underwent exercise stress test before and after dantrolene infusion. Dantrolene reduced the number of premature ventricular complexes (PVCs) on average by 74% (range 33-97) in four patients with N-terminal or central mutations in the cytosolic region of the *RyR2* protein, while dantrolene had no effect in two patients with mutations in or near the transmembrane domain. Induced pluripotent stem cells (iPSCs) were generated from all the patients and differentiated into spontaneously beating cardiomyocytes (CMs). The antiarrhythmic effect of dantrolene was studied in CMs after adrenaline stimulation by Ca^2+ ^ imaging. In iPSC derived CMs with *RyR2* mutations in the N-terminal or central region, dantrolene suppressed the Ca^2+^ cycling abnormalities in 80% (range 65-97) of cells while with mutations in or near the transmembrane domain only in 23 or 32% of cells. In conclusion, we demonstrate that dantrolene given intravenously shows antiarrhythmic effects in a portion of CPVT1 patients and that iPSC derived CM models replicate these individual drug responses. These findings illustrate the potential of iPSC models to individualize drug therapy of inherited diseases.

**Trial Registration:**

EudraCT Clinical Trial Registry 2012-005292-14

## Introduction

Catecholaminergic polymorphic ventricular tachycardia (CPVT) is one of the most malignant inherited arrhythmogenic disorders. It manifests with exercise-induced premature ventricular complexes (PVCs), polymorphic or bidirectional ventricular tachycardia, or sudden death, usually associated with vigorous physical exercise or mental stress.[1–3] Current therapeutic options include beta-antiadrenergic drugs, flecainide, implantable cardioverter-defibrillators (ICD) [4–6] and left cardiac sympathetic denervation.[7,8] Better antiarrhythmic medication is still needed to minimize the need for ICD shock therapies. The most common subtype, type 1 of CPVT (CPVT1) is a dominantly inherited disease caused by mutations in the cardiac ryanodine receptor (*RyR2*) gene.[9,10] The gain-of-function mutations of *RyR2* cause increased calcium (Ca^2+^) sensitivity which can lead to spontaneous Ca^2+^ release from sarcoplasmic reticulum, generation of afterdepolarizations, and triggered activity.[4,5,11]

The ryanodine receptor isoform *RyR1* is the skeletal muscle counterpart in the gene family. Mutations of *RyR1* result in malignant hyperthermia, a rare but life-threatening complication of general anesthesia occurring upon administration of volatile anesthetics or depolarizing muscle relaxants. Dantrolene is a specific and currently the only effective treatment for malignant hyperthermia.[12] Interestingly, dantrolene has also shown to exert antiarrhythmic effects in animal models of CPVT1.[13–15] Dantrolene has been proposed to act through binding to the N-terminal parts of *RyR1* and *RyR2* and restoring inter-domain interactions critical for the closed state of the *RyR2* Ca^2+^ channel.[16]

Several studies using induced pluripotent stem cell (iPSC) technology [17] have indicated the ability of CPVT1 patient-specific iPSC derived cardiomyocytes (CMs) to replicate the disease phenotype in cell culture.[18–24] Dantrolene was reported to rescue the disease phenotype in iPSCs derived CMs from a single *RyR2* mutation carrier [20] but no *in vivo* data exists on its effects on CPVT1 patients.

In the present study, we report the proof of principle of the antiarrhythmic activity of dantrolene in a cohort of CPVT1 patients. In addition, we demonstrate that the in vivo drug effects are closely reproduced in iPSC-derived patient-specific CMs, and provide evidence for mutation-specific effects of dantrolene in CPVT1.

## Materials and Methods

The protocol for this trial is available as supporting information; see S1 Protocol and S2 Protocol.

### Clinical Study Scheme

The study was approved by the Ethical Review Committee of the Helsinki University Hospital (HUS 396/13/03/01/12) and was in accordance with the institutional guidelines and the Declaration of Helsinki. A written informed consent was obtained from all patients. Clinical trial was registered with EudraCT (2012-005292-14). Participants were recruited between 1^st^ March 2013 and 29^th^ May 2014. Follow up time of the patients was three days after the dantrolene infusion. Four patients participated the study at the Helsinki University Hospital and two at the Tampere University Hospital, Heart Center. The authors confirm that all ongoing and related trials for this drug are registered.

### Patients

**Fig 1:**
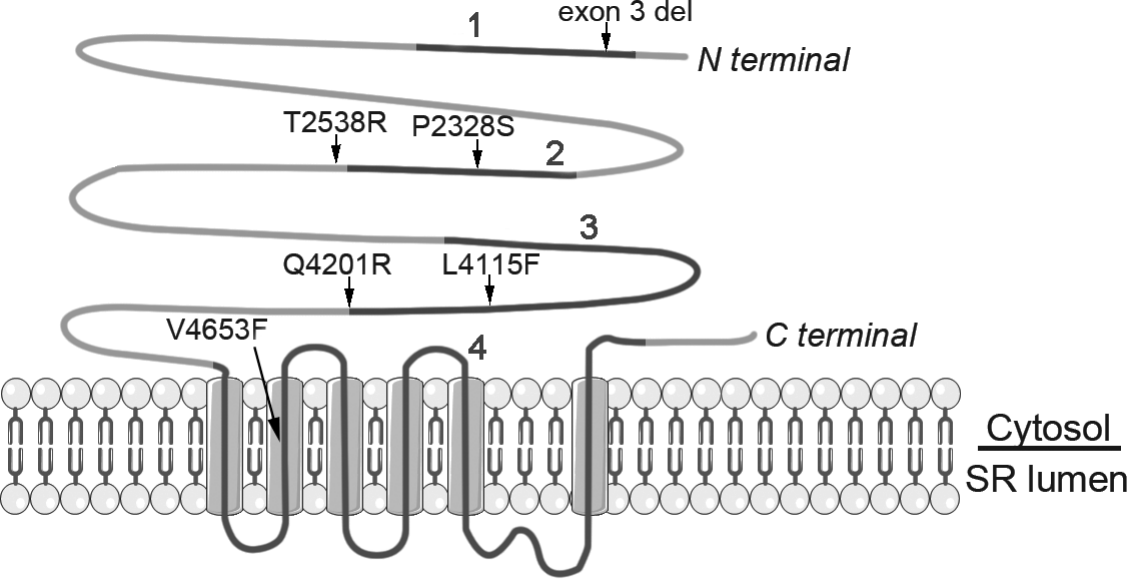
*RyR2* protein, mutations studied in the present study and mutation clusters 1–4. Mutations in this study (arrows) are located in different parts of the *RyR2* protein and mutation clusters. Clusters are represented as black lines numbered from 1 to 4. Cluster 1 comprises of amino acids (AA) 44–466, cluster 2 AA 2246–2534 and cluster 3 AA 3778–4201 and these three clusters are located in the N-terminal and central regions of the protein and form the cytoplasmic domain. Cluster 4 comprises of AA 4497–4959 and forms the transmembrane domain, which is located in the C-terminal region. The figure is modified from [11].

The study group consisted of 6 individuals (mean age 50±10 years, range 37–59 years, 5 females), who were molecularly defined heterozygous carriers of different gain-of-function *RyR2* mutations causing CPVT1. CPVT1 patients carried the following mutations: *c*.*168-301_c*.*273+722del1128* mutation (later called as *exon 3 deletion)* or point mutations *p*.*P2328S (c*.*6982C>T)*, *p*.*T2538R (c*.*7613C>G)*, *p*.*L4115F (c*.*12343C>T)*, *p*.*Q4201R (c*.*12602A>G)* or *p*.*V4653F (c*.*13957G>T)*. Mutation nomenclature was based on *RyR2* reference sequence NM_001035.2. The mutations were located in the four mutation hotspot clusters of the *RyR2* gene (Fig 1).[11] Patients and families carrying mutations *P2328S* [3,10,25], *exon 3 deletio*n [3,26], *Q4201R* [10,25] and *V4653F* [10,25] have been described in detail earlier.

All mutations were associated with exercise-induced ventricular arrhythmias and syncopal spells. All except *L4115F* were associated with one or more cases of sudden death at young age in the family. An ICD had been implanted in five of them; one has received an adequate shock therapy. Five out of the six patients had a history of syncopal spells upon a frightening situation or physical exercise, and they all used a beta-adrenergic blocking agent (daily dose of 160 mg of propranolol, 7, 5 to 10 mg of bisoprolol or 95 mg of metoprolol) during all study phases. No other medications were in use. The patients were otherwise healthy without hypertension, diabetes, or evidence of other heart disease. None of them had bundle branch block.

In cardiac ultrasonography, left ventricular end diastolic dimension and systolic function were normal in all patients (data not shown). Basic laboratory parameters including hemoglobin, white blood cell count, plasma sodium, potassium, and creatinine concentrations were analyzed prior to administration of the drug and they were all within the normal range in every study patient (data not shown). Two of the patients with ICD showed atrial pacing at rest prior to exercise; all others had sinus rhythm. Electrocardiographic parameters are presented in Table 1.

**Table 1:** Electrocardiographic parameters.

	Before dantrolene (n = 6)	After dantrolene (n = 6)	p-value
**Heart rate (min-1)**	61±5	60±5	NS
**P (ms)**	109±14	110±14	NS
**PQ (ms)**	154±31	163±34	NS
**QRS (ms)**	86±8	87±5	NS
**QT (ms)**	419±22	407±18	NS
**QTc (ms)**	418±35	409±35	NS

Comparison of electrocardiographic parameters of all patients before and after the dantrolene infusion. NS indicates no significant.

### Clinical Exercise Stress Test

All RyR2 patients underwent the exercise stress test three times. On the first morning a baseline study was carried out. The test was repeated in the afternoon of the first day after intravenous infusion of dantrolene sodium (Dantrium, 1.5 mg per kg of body weight). The third exercise test was performed on the second day to assess the effects after dantrolene sodium washout and to demonstrate the reproducibility of the basic exercise test.

Exercise tests were performed with a bicycle ergometer. The initial load was 30 W, followed by increments of the load by 15 W each minute. In the baseline study, the patient was instructed to target to a submaximal workload in order to be able to repeat the exercise stress test. In the consecutive phases of the study, the workload target was the same as that achieved in the first study. A twelve-lead electrocardiogram (ECG) was recorded continuously at paper speed 25 or 50 mms^-1^ and amplification of 0.1 mV/mm throughout the exercise test. After cessation of exercise, ECG was recorded continuously for the first 8 minutes. Both the maximum workload achieved and the heart rate at which ventricular bigeminy first appeared were recorded whenever applicable. Numbers of PVCs during exercise and at recovery phase as well as the maximum number of consecutive PVCs were counted. Plasma creatinine, sodium, potassium and calcium were measured at rest before the first exercise test. The exercise tests and the iPSC studies were done separately and blinded.

### Characterization of iPSC Lines

The iPSC study was approved by the ethical committee of Pirkanmaa Hospital District (R08070) and written informed consent was obtained from all the participants. Patient-specific iPSC lines were established as described earlier.[17] Studied iPSC lines were UTA.05605.CPVT generated from patient with *RyR2 exon 3 deletion*, UTA.05208.CPVT from patient with mutation *P2328S*, UTA.07001.CPVT from patient with mutation *T2538R*, UTA.03701.CPVT from patient with mutation *L4115F*, UTA.05503.CPVT from patient with mutation *Q4201R*, UTA.05404.CPVT from patient with mutation *V4653F* and UTA.04602.WT from a healthy control individual.

All the CPVT-iPSC lines were characterized for their karyotypes, mutations, pluripotency, immunocytochemistry, embryoid body (EB) and teratoma formation. Endogenous and exogenous gene expressions were examined by RT-PCR using 1 μl cDNA and 500 nmol/L of each primer in one PCR reaction. β-actin and GAPDH served as the housekeeping genes. Detailed reaction conditions and PCR primers for iPSC characterization have been described earlier.[27] Endogenous pluripotency markers at the protein level were studied with immunocytochemistry. The iPSCs were fixed with 4% paraformaldehyde (PFA, Sigma-Aldrich, Saint Louis, USA). Primary antibodies anti-SOX2, anti-NANOG and anti-tumor-related antigen (TRA)1-81 (all 1:200, from Santa Cruz Biotechnology, Santa Cruz, CA, USA) and anti-OCT3/4 (1:400, R&D Systems) were used. Cells were mounted with Vectashield (Vector Laboratories, USA) containing DAPI for staining nuclei. To confirm the mutations of the CPVT iPSC lines with sequencing the DNA was isolated using DNA Tissue XS-kit (Macherey-Nagel GmbH & Co., Düren, Germany). The genomic region containing the expected mutation was amplified using PCR. Each PCR product was directly sequenced in both directions using BigDye Terminator v3.1 and ABI 3730xl DNA Analyzer (Applied Biosystems, Carlsbad, CA, USA). In addition to direct sequencing, *exon 3 deletion* (1128 nucleotides) was also confirmed using PCR and agarose gel electrophoresis. Karyotypes of the cell lines were determined using either standard G-banding chromosome analysis (Medix laboratories, Espoo, Finland) or KaryoLite BoBs assay (Perkin Elmer) based on BACs-on-Beads technology (Molecular and Systems Immunology and Stem Cell Biology, Turku Centre for Biotechnology, University of Turku, Finland). The expression of markers characteristic of ectoderm (*Nestin* or *SOX-1*), endoderm (*AFP* or *SOX-17*), and mesoderm (*VEGF-R2*) development were studied from EBs maintained in EB-medium (KO-DMEM with 20% FBS, Non-Essential Amino Acid (NEAA), L-glutamine and penicillin/streptomycin) for 5 weeks. EB RT-PCR primers can be seen in S1 Table. The teratoma study was approved by ELLA- Animal Experiment Board of Regional State Administrative Agency for Southern Finland (ESAVI/6543/04.10.03/2011). IPSCs were injected into nude mice under the testis capsule and tumor samples collected 8 weeks after injection, followed by fixation with 4% PFA and staining of the sections with hematoxylin and eosin.

### Cardiomyocyte Differentiation and Characterization

iPSCs were co-cultured with murine visceral endoderm-like (END-2) cells (Humbrecht Institute, Utrecht, The Netherlands) to differentiate them into spontaneously beating CMs. The beating areas of the cell colonies were mechanically excised and treated with collagenase A (Roche Diagnostics).[28] Single CMs were immunostained with anti-cardiac-troponin-T (1:1500, Abcam, Cambridge, MA, USA), anti-α-actinin (1:1500, Sigma) and anti-connexin-43 (1:1000, Sigma).

### Ca^2+^ Imaging

Dissociated spontaneously beating CMs on a coverslip were loaded with 4 μmol/L Fura 2-AM (Life Technologies, Molecular Probes). CMs were continuously perfused with 37°C HEPES based perfusate during measurements and the perfusate consisted of (in mmol/L): 137 NaCl, 5 KCl, 0.44 KH_2_PO_4_, 20 HEPES, 4.2 NaHCO_3_, 5 D-glucose, 2 CaCl_2_, 1.2 MgCl_2_ and 1 Na-puryvate (pH was adjusted to 7.4 with NaOH). Ca^2+^ measurements were conducted on an inverted IX70 microscope (Olympus Corporation, Hamburg, Germany) and cells were visualized with UApo/340 x20 air objective (Olympus). Images were acquired with an ANDOR iXon 885 CCD camera (Andor Technology, Belfast, Northern Ireland) synchronized with a Polychrome V light source by a real time DSP control unit and TILLvisION or Live Acquisition software (TILL Photonics, Munich, Germany). Fura 2-AM in CMs was excited at 340 nm and 380 nm light and the emission was recorded at 505 nm. For Ca^2+^ analysis, regions of interest were selected for spontaneously beating cells and background noise was subtracted before further data processing. The Ca^2+^ levels are presented as fura ratio units of F340/F380. Ca^2+^ peaks were analyzed with Clampfit version 10.2 (Molecular Devices, USA).

The percentage of abnormal Ca^2+^ transients, such as multiple peaks comprising of two peaks, irregular phases, oscillations, and varying amplitude manifested as low peaks, were calculated from each studied cell line. Beating frequency and diastolic Ca^2+^ levels of CMs were analyzed during spontaneous baseline beating, and during adrenaline perfusion. These parameters were compared between mutated and control cell lines and also between each mutated cell line. Some results of Ca^2+^ cycling of CPVT-*P2328S* and control cell lines have been published before [21] and parts of these results have been included here.

For dantrolene studies, the changes in Ca^2+^ were recorded during spontaneous baseline beating, spontaneous beating during 1 μM adrenaline perfusion and spontaneous beating during 1 μM adrenaline together with 10 μM dantrolene (Sigma) perfusion. If a CM displayed Ca^2+^ transient abnormalities during adrenaline perfusion, it was exposed to dantrolene. Drug effects of dantrolene were categorized into three groups. In “responder” group dantrolene abolished virtually all the Ca^2+^ handling abnormalities, in “semi-responder” group dantrolene reduced them by more than 50%, in “non-responder” group dantrolene reduced them by less than 50%. Diastolic Ca^2+^ levels and beating frequency were compared between adrenaline and dantrolene responses of responder CMs (*exon 3 del*, *P2328S*, *T2538R*, *L4115F*) and non-responder CMs (*Q4201R*, *V4653F*, controls). For this, adrenaline response values were divided by dantrolene response values, separately for each cell.

### Statistical Analysis

Statistical analysis of *in vivo* studies was made with SPSS 21.0 statistical software package (SPSS, Chicago, IL). Data are presented as average + 1 SD. Comparisons between phases were performed by the non-parametric Wilcoxon test. The significance of *in vitro* differences between two groups was evaluated with the unpaired Student’s *t*-test. The significance of changes within a group was evaluated with the paired Student’s *t*-test. Data are expressed as average ± S.E.M. and n refers to the number of cells. P<0.05 was considered statistically significant in both *in vivo* and *in vitro*.

## Results

### Antiarrhythmic Effects of Dantrolene in Cpvt1 Patients

In the baseline study, patients exercised on an average 8±2 minutes reaching a maximum heart rate of 134±17 min^-1^. Exercise bicycle testing induced polymorphic PVCs in all patients and non-sustained ventricular tachycardia (NSVT, episodes of 3 to 4 consecutive PVCs) in three of them. The average threshold sinus rate for the appearance of PVCs was 105±9 min^-1^. The total count of PVCs during the workload was 172±119 (range 43–391).

**Fig 2:**
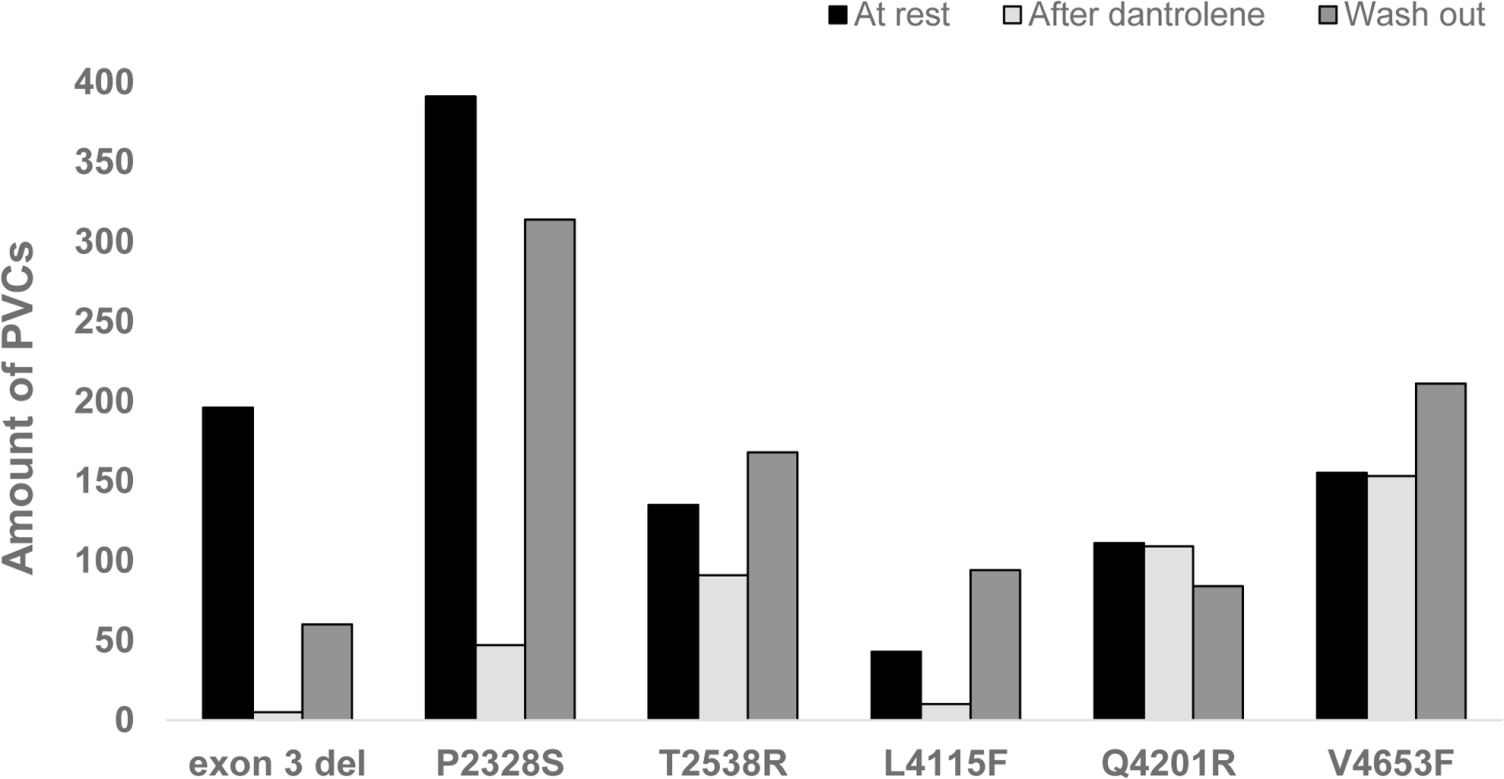
Features of the PVCs. Number of PVCs in exercise stress test before and after administration of intravenous dantrolene and 24 hours after dantrolene wash out.

All six patients tolerated the target dose 1.5 mg/kg of intravenous infusion of dantrolene but reported considerable muscle weakness as a side-effect. Dantrolene did not affect atrioventricular conduction or the QT interval (Table 1). Dantrolene decreased the prevalence of PVCs in four patients, whereas in two patients the number of PVCs remained virtually the same (Fig 2). Dantrolene seemed to reduce arrhythmias in patients with the mutation in the N-terminal or central region of the *RyR2* protein (Figs 1 and 2). Thus, dantrolene abolished 97% of PVCs in the patient with exon 3 deletion (cluster 1), 88% of PVCs in the patient with *P2328S* mutation (cluster 2), 33% of PVSc in the patient with *T2538R* mutation (right after cluster 2) and 77% of PVCs in the patient with *L4115F* mutation (cluster 3). In contrast, dantrolene abolished only 1 to 2% of PVCs in patients carrying mutation closer to (*Q4201R*, end of cluster 3) or within the transmembrane region (*V4653F*, cluster 4).

**Fig 3:**
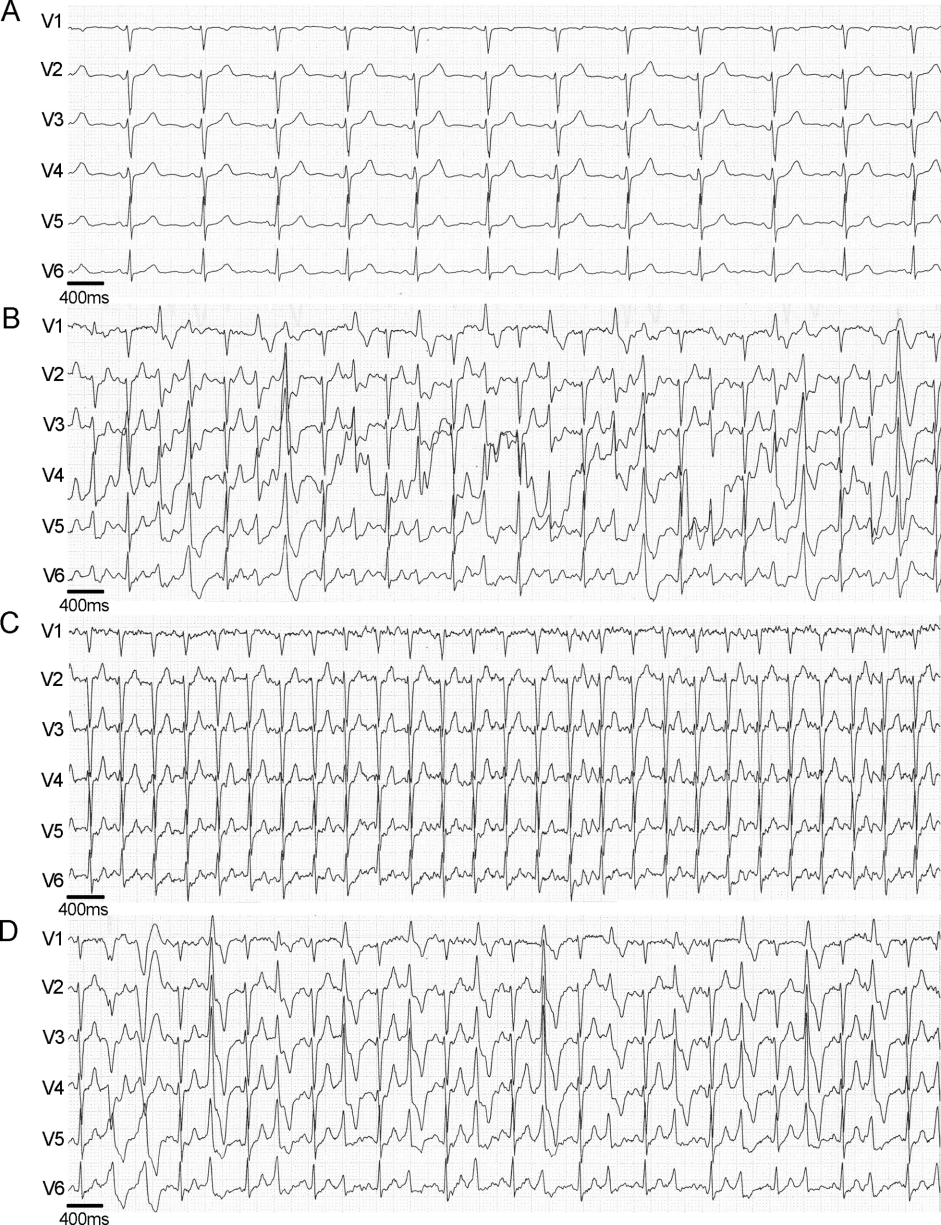
ECG examples of a 38-year-old patient carrying the *RyR2 P2328S* mutation. (A) Resting ECG showing sinus rhythm and normal QRS morphology. (B) Exercise ECG at the highest work load of 105 W in the baseline study before dantrolene. PVCs include couplets and polymorphic NSVTs. (C) Disappearance of ventricular arrhythmias after administration of dantrolene (work load 105 W). (D) Exercise test on day two after 20-hours wash-out of dantrolene showing return of PVCs (work load 105 W).

Fig 3 illustrates an example of the PVCs and NSVT episodes during the baseline study and after dantrolene. Dantrolene increased significantly the threshold at which the arrhythmias appeared from 105±9 to 120±17 min-1. The duration of the exercise phase and the maximal heart rate achieved during the exercise were similar to those in the baseline study. On day 2, after wash-out of dantrolene, the prevalence of PVCs was approaching that in the first baseline test.

### Characterization of iPSC Lines Confirms Pluripotent Stem Cell Characteristics

**Fig 4:**
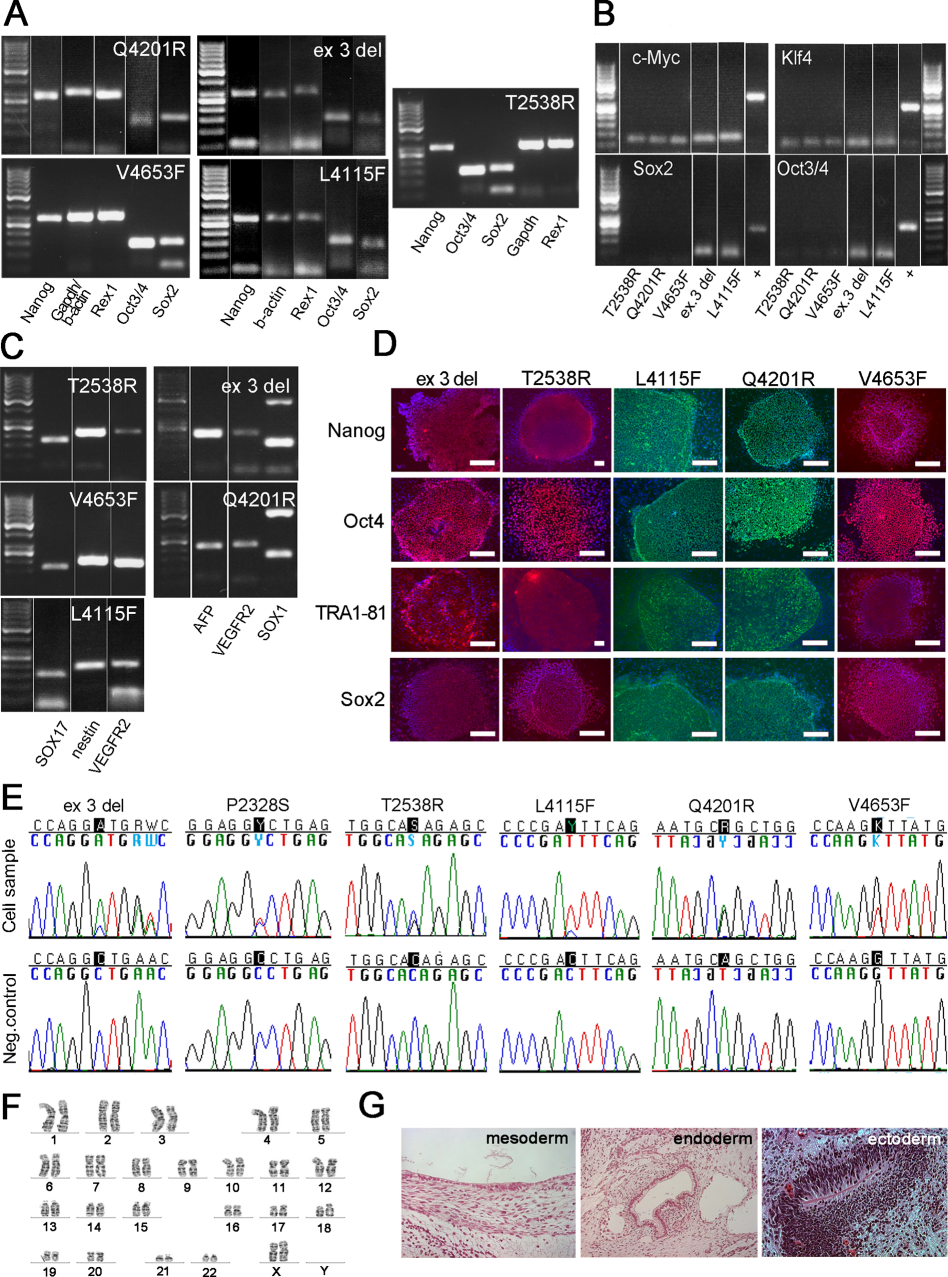
Characterization of CPVT1 iPSCs. (A) Expression of pluripotency markers shown by RT-PCR, β-actin or GAPDH serving as a housekeeping gene. (B) None of the exogenous genes are expressed in CPVT1 cell lines. (C) EBs express markers from all the three embryonic germ layers. (D) Immunocytochemical stainings and expression of pluripotency markers. Scale bar 200 μm. (E) Sequencing analysis confirmed the *RyR2* mutation in each cell line. (F) All the cell lines had normal karyotype, example picture from *Q4201R* cell line. (G) Teratomas made from a CPVT-iPSC line further confirms pluripotency, example pictures from *L4115F* cell line.

**Fig 5:**
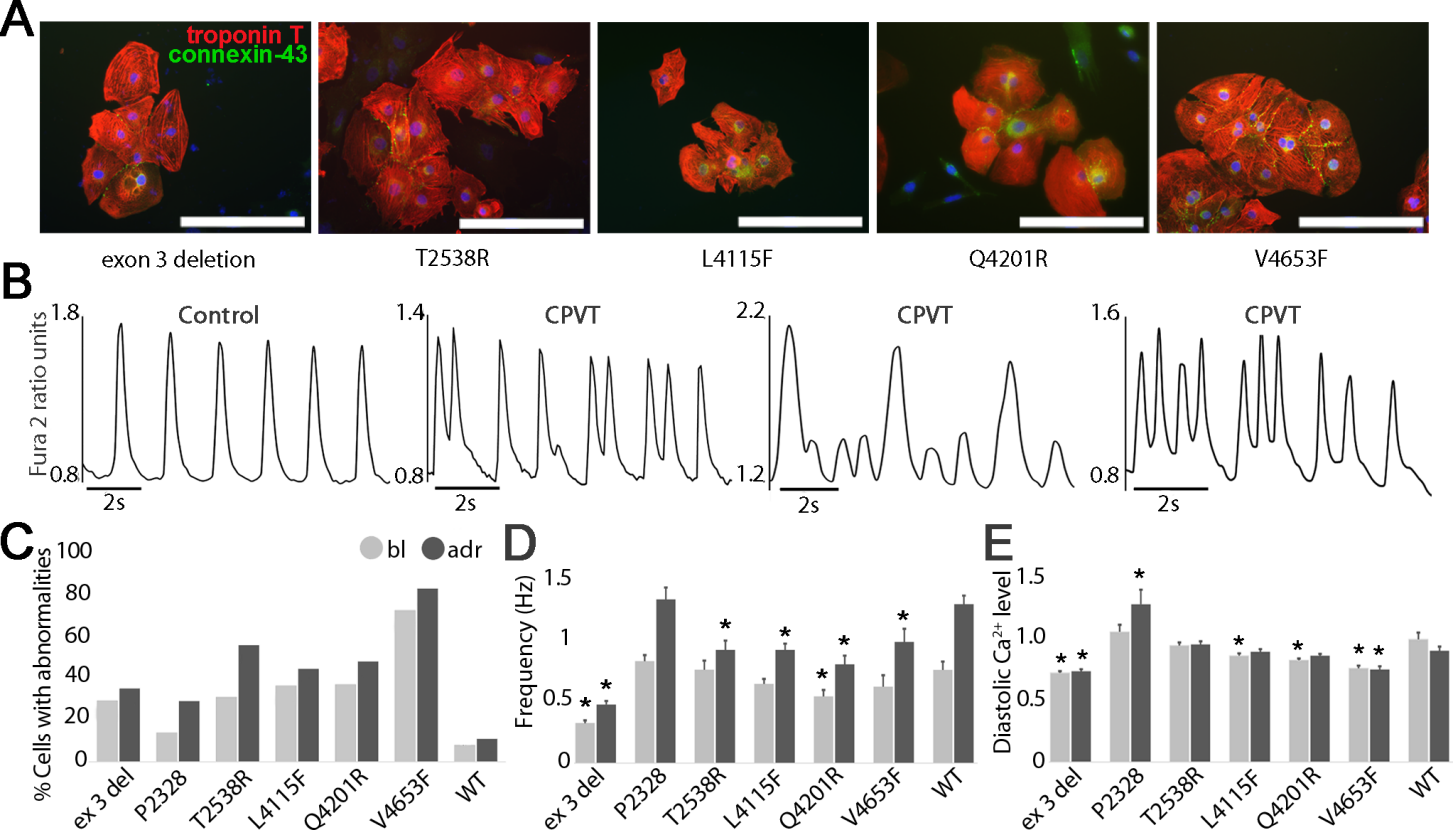
Characterization of CPVT-iPSCs derived CMs. (A) Immunocytochemical stainings of cardiac markers where red represents troponin T, green connexin-43 and blue DAPI-staining for nuclei. Scale bars 200 μm. (B) Representative traces of a control CM showing normal regular Ca^2+^ transients and CPVT1 CMs showing abnormalities like multiple peaks, low peaks, irregular phases and oscillations in Ca^2+^ handling. (C) Quantification of percentage of CPVT1 and control iPSC CMs exhibiting abnormal Ca^2+^ transients at baseline (bl) and during adrenaline perfusion (adr). (D) Frequency and (E) Diastolic level of intracellular Ca^2+^ of all CPVT1 and control CMs. Numbers of cells analyzed in C, D, and E, *exon 3 del* n = 48, *P2328S* n = 72, *T2538R* n = 52, *L4115F* n = 110, *Q4201R* n = 63, *V4653F* n = 29, Controls (WT) n = 28. As an exception, number of WT cells analyzed in D, and E, in bl n = 54 and adr n = 27 and number of *P2328S* cells in bl n = 90 and adr n = 47. Grey bars indicate cells at baseline and black bars during adrenaline perfusion. Error bars, SEM. *P<0.05 CPVT1 versus control, with Student’s t-test. Significance’s of mutation specific differences, see S2 Table.

iPSC lines from six CPVT1 patients with above-mentioned *RyR2* mutations were generated. Results of the *P2328S* and control iPSC line characterizations have been published before.[21,27] All studied endogenous pluripotency genes (Nanog, Rex1, Oct 3/4 and Sox2) were turned on and the expression of retrovirally encoded reprogramming factors c-Myc, Klf4, Sox2 and Oct 3/4 was silenced (Fig 4A and 4B). CPVT1 iPSC lines expressed endogenous pluripotent markers Nanog, Oct3/4, TRA 1–81 and SOX2 also at the protein level (Fig 4D). Pluripotency was further confirmed by teratoma formation and with *in vitro* embryoid body (EB) formation expressing all three germ layers (Figs 4C and 5G). The presence of the *RyR2* mutations was confirmed from all the CPVT1 iPSC lines with DNA sequence analysis (Fig 4E). All the iPSC lines had a normal karyotype (Fig 4F). In addition to sequencing, the presence of the *exon 3 deletion* was confirmed with PCR (S1 Fig).

### iPSC Derived Cms Display Abnormal Ca^2+^ Cycling

iPSCs were differentiated into spontaneously beating CMs (Fig 5A). When compared to CMs derived from the healthy individual, CPVT1 CMs demonstrated marked Ca^2+^ transient abnormalities such as multiple peaks comprising of two peaks, irregular phases, oscillations, and varying amplitude manifested as low peaks (Fig 5B) both in baseline and in response to adrenaline. Although these abnormalities were common with all six mutations examined, some differences between *RyR2* mutations were also observed. Accordingly, Ca^2+^ transient abnormalities were somewhat more common in the cluster 4 mutation than in cluster 1, 2 and 3 mutations (Fig 5C).

Adrenaline increased the beating frequency of each cell line studied (Fig 5D). All *RyR2* mutated CMs had lower beating frequency both at baseline and during adrenaline perfusion than control CMs. Adrenaline produced significantly elevated diastolic Ca^2+^ levels only in *P2328S* CMs, while diastolic Ca^2+^ levels were lower or similar in other mutant CMs compared to control CMs (Fig 5E). *Exon 3 deletion* CMs had both lower beating frequency and diastolic Ca^2+^ level when compared to other mutations (Fig 5 and S2 Table).

### iPSC Derived Cpvt1 Cms Reproduced the Clinical Antiarrhythmic Responses to Dantrolene

**Fig 6:**
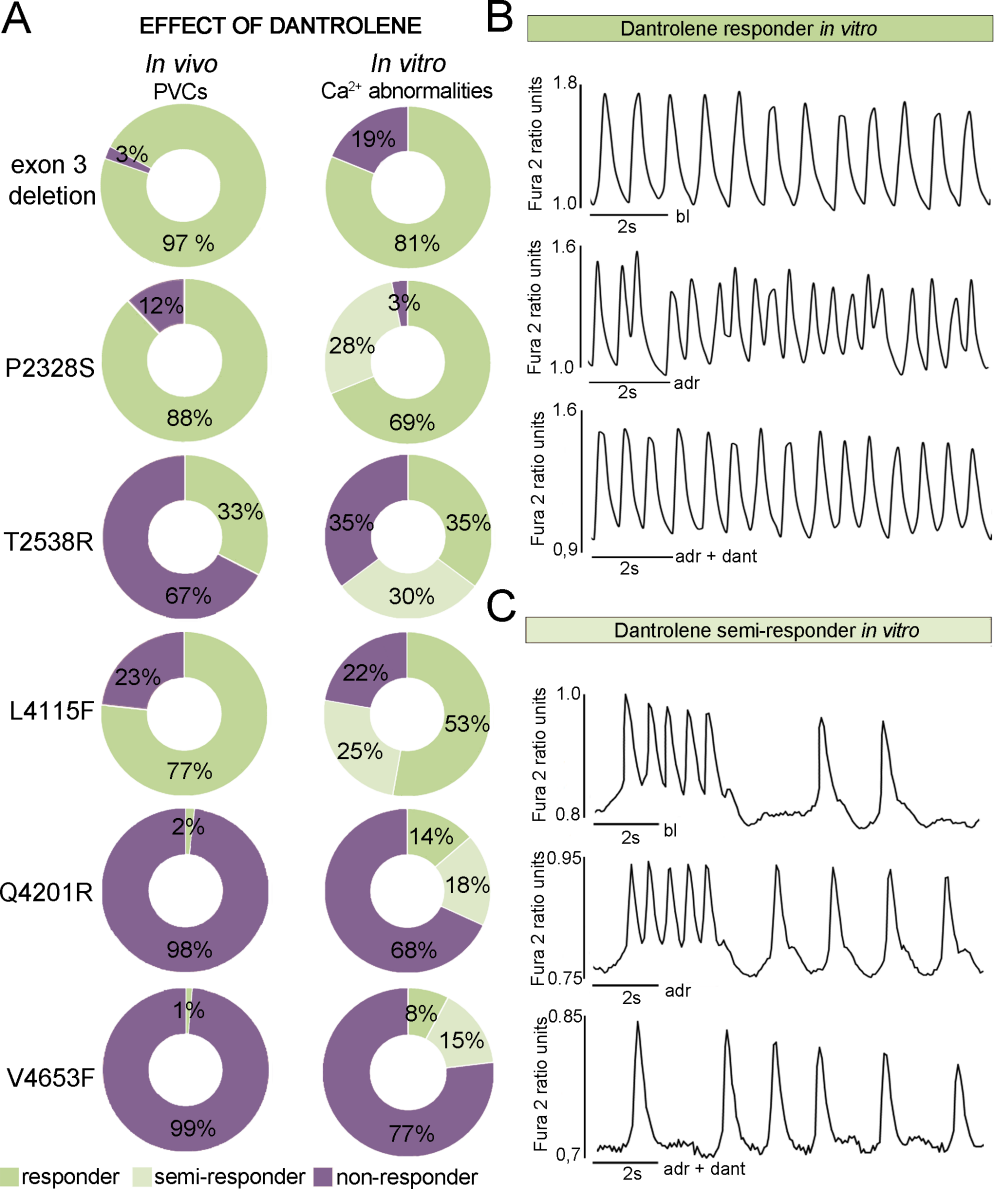
iPSC derived CMs reproduced the clinical responses of dantrolene. (A) *In vivo* and *in vitro* effects of dantrolene correspond within each *RyR2* mutation. *In vitro* drug effects were categorized into three groups (responders, semi-responders and non-responders) depending on how dantrolene affected to the amount of Ca^2+^ abnormalities when compared to adrenaline response. *In vivo* responder group show the percentage of the abolished PVCs when compared to the baseline. Numbers of cells analyzed in *exon 3 del* n = 16, *P2328S* n = 32, *T2538R* n = 17, *L4115F* n = 36, *Q4201R* n = 22, *V4653F* n = 13. (B) Representative traces of dantrolene responder *in vitro* in an *L4115F* mutated CM. Adrenaline causes Ca^2+^ cycling abnormalities and dantrolene abolishes all the abnormalities. (C) Representative traces of semi-responder *in vitro* in a *P2328S* mutated CM. The cell has abnormal Ca^2+^ cycling at baseline and during adrenaline perfusion and dantrolene reduces the abnormalities by abolishing the oscillation but leaving some low peak Ca^2+^ spiking.

Effects of dantrolene were divided into three groups based on their Ca^2+^ responses. In “responder” group dantrolene abolished all the Ca^2+^ handling abnormalities, in “semi-responder” group dantrolene reduced them by more than 50% and in “non-responder” group dantrolene reduced them by less than 50%. iPSC derived CMs were found to markedly reproduce the varying individual clinical responses of dantrolene (Fig 6). In cell lines with the mutation in the N terminal or central region of the *RyR2* protein dantrolene abolished or reduced the majority of Ca^2+^ transient abnormalities (Fig 6). These mutations were within or in close proximity of clusters 1, 2 or 3 (Fig 1). A detailed analysis indicated that in CMs with *exon 3 deletion*, *P2328S*, *T2538R or L4115F*, dantrolene abolished or reduced by more than 50% of Ca^2+^ abnormalities in 65–97% of cells (Fig 6).

In striking contrast, the effect of dantrolene was only minimal in CMs carrying a mutation at the end of cluster 3 (*Q4201R*) or in the transmembrane region (cluster 4, mutation *V4653F*) (Fig 6), in accordance with the *in vivo* dantrolene infusion data. Dantrolene had no effect on the Ca^2+^ transients in control CMs. Dantrolene did not significantly affect the diastolic Ca^2+^ levels of CMs in which Ca^2+^ transient abnormalities were abolished (S2 Fig). Dantrolene increased significantly the diastolic Ca^2+^ levels of control and *Q4201R* CMs where Ca^2+^ transients were unaltered by the drug. There was no correlation between the antiarrhythmic effect of dantrolene and its effect on beating frequency (S2 Fig).

## Discussion

We have studied the antiarrhythmic potential of dantrolene in the treatment of CPVT1. To this end, we assessed the efficacy of intravenously administered dantrolene in patients carrying various *RyR2* mutations and compared these effects to *in vitro* studies using iPSC derived CMs generated from the same patients. Our findings demonstrate that intravenous dantrolene, a drug used to treat another ryanodine receptor disorder, malignant hyperthermia, abolished or markedly reduced arrhythmias in a subgroup of CPVT1 patients with specific *RyR2* mutations. By combining evidence from *in vivo* and *in vitro* studies, we propose that the location of the *RyR2* mutation affects the antiarrhythmic effect of dantrolene in CPVT1.

Previously dantrolene has been shown to have beneficial effects on cardiac function in experimental animal models of CPVT1 [13–15,29,30] and in iPSC derived CMs from a CPVT1 patient with an N-terminal *S406L* mutation [20], but no studies in patients have so far been reported. It is also important to take into consideration that even if a drug is found to be beneficial in the patient-derived iPSC-CMs, it cannot be automatically concluded that this will translate into a clinical benefit. Here we show that dantrolene given intravenously has an antiarrhythmic effect also in some but not in all patients with CPVT1. This antiarrhythmic effect was observed only in patients with *RyR2* mutations in the N-terminal or central regions of *RyR2* protein (clusters 1–3), whereas virtually no effect was seen in patients carrying mutations at the end of cluster 3 or in the transmembrane region (cluster 4). Although a dose-dependent effect cannot be excluded, similar observations on mutation-specific drug responses have been obtained in some other genetic disorders including long QT syndrome type 3 [31], cystic fibrosis [32], as well as in certain neoplastic diseases.[33] Recognition of potential mutation-specific responses will be important for future drug development: one drug may not work for all patients even if the phenotype is the same.

Currently, beta-antiadrenergic medication is the first line antiarrhythmic treatment for all CPVT patients. Flecainide has also shown beneficial effects.[34,35] ICDs are used if severe arrhythmic events occur despite optimal beta-blocking treatment. However, use of ICDs is not without risk since ICD-shocks may further aggravate catecholamine release and initiate an uncontrolled electric storm. Very recent data suggest that left cardiac sympatectomy may be highly effective in patients refractory to medical therapy.[7,36] Although dantrolene as such would not be suitable for long-term treatment of CPVT1 due to its side effects, and although only a subset of patients would benefit from it, our data shows its antiarrhythmic potential. It also suggest that it could be administered by intravenously to CPVT1 patients in emergencies such as incessant ventricular tachycardia.

More than 150 mutations in *RyR2* gene have been reported so far and they are clustered in four hotspots.[11] One third of the reported mutations are in clusters 1 and 2 and the rest are equally distributed between clusters 3 and 4. Only 10% of *RyR2* mutations have been found outside these clusters.[11] The location of the *RyR2* mutation appears to be critical for a favorable effect of dantrolene. The binding site for dantrolene is localized in the N-terminus of *RyR2* between amino acid 601 and 620.[13,37] The dantrolene-binding sequence is considered to constitute part of the domain switch region, suggesting that dantrolene is involved in the correction of defective unzipping and allosteric stabilization of interdomain interactions between the N- terminal and central regions of *RyR2*, resulting in inhibition of Ca^2+^ leak [16,30,38] and in fact, this has been demonstrated in previous studies.[13,16,37] Also our data demonstrate that dantrolene abolished arrhythmias in CPVT1 patients with mutations of N terminal or central domain, suggesting that a defective inter-domain interaction within the *RyR2* could be the underlying arrhythmogenic mechanism in the *exon 3 deletion*, *P2328S*, *T2538R* and *L4115F*. However, dantrolene did not suppress *T2538R*-related arrhythmias to the same extent as arrhythmias caused by other central region mutations. It has been speculated that differences in the mode of interdomain interaction in dantrolene binding regions may result in differences in its antiarrhythmic efficacy.[30] Furthermore, other drug-binding regions in the carboxyl-terminal half of the *RyR2* or additional low affinity drug binding sites in the N-terminal area could exist.[13] No previous studies on the effects of dantrolene on *RyR2* mutations in or close to the transmembrane are available; here we show that dantrolene has no or only minimal effect on arrhythmias if mutations are located in these areas. It is interesting that the patient with *Q4201R* mutation did not respond to dantrolene even though this mutation is located in cytosolic portion of *RyR2* and in cluster 3 although in its terminal part. This finding indicates that the location of the mutation in certain mutation cluster does not necessarily determine the antiarrhythmic response, and highlights the utility of the iPSC model for individual functional analysis.

Our data showing similar patient-to-patient variation in dantrolene effects in the clinical setting and corresponding iPSC-CM models suggest that, at least in theory, it may be possible to tailor an individual's medication in cell culture without predisposing the individual to the potentially serious side-effects of a drug. Dantrolene did not affect normally beating CMs. This is consistent with previous reports showing that dantrolene inhibits only abnormal Ca^2+^ release and has no effect on the normal Ca^2+^ transients, suggesting that the native conformation of *RyR2* may restrict binding of the drug and that dantrolene binding to *RyR2* might be dependent on a specific conformational state present only in mutated cells.[13,37] Defective calmodulin binding caused by *RyR2* domain unzipping has also been shown to be restored by dantrolene [29,39] which may as well explain why dantrolene exerts effects on diseased but not healthy hearts.

Besides differences in drug responses, we also saw both similarities and differences in the CPVT1 *in vitro* phenotypes depending on the nature of the mutation. The beating frequency of CPVT1 CMs was lower than that in control CMs. This is in line what has been reported also with CPVT1 patients.[5,40] All the CPVT1 CMs showed similar disturbances in intracellular Ca^2+^ cycling. Ca^2+^ transient abnormalities were somewhat more common in the cluster 4 mutation than in cluster 1, 2 and 3 mutations. *Exon 3 deletion* differed from all the other mutations by having lower diastolic Ca^2+^ levels and beating frequency both at baseline and during adrenaline perfusion. Exon 3 encodes secondary structure elements that are crucial for folding of the N-terminal domain. It has been proposed that *RyR2* with *exon 3 deletion* has evolved additional means to regulate Ca^2+^ release, by altering the conformation of the domain [41], which may result in the observed differences in Ca^2+^ transients.

There are certain limitations in our study. First, only six CPVT1 patients and their iPSC cell lines were studied; however, we emphasize that despite the very rare nature of this disease we were able to examine in detail six different disease-causing mutations. Second, under the conditions of the study design we were permitted to study only acute effects of intravenously administered dantrolene. Third, although we titrated the dose of dantrolene according to the weights of the patients, serum levels of the drug were not measured and could have varied from patient to patient, resulting in concentration-dependent variation in clinical responses. Fourth, we used only a fixed concentration of dantrolene, selected on the basis of the work by Jung et al. [20], in our iPSC studies. Fifth, immature phenotype of the iPSC-CMs may produce variation in arrhythmias. However, in our previous study [21] the electrophysiology of CPVT1 iPSC-CMs appeared fairly mature. We have also shown here and in our previous study [21] that arrhythmias are substantially more consistent in CPVT1 CMs than in control CMs.

In conclusion, we have shown here the proof of principle that intravenously administered dantrolene suppresses ventricular arrhythmias in the congenital *RyR2* defect and that the location of the *RyR2* mutation may affect the antiarrhythmic effect of this drug. We also demonstrate that iPSC derived patient-specific CMs correctly predict the clinical response to dantrolene in CPVT1 patients with varying *RyR2* mutations. Our data support the notion that iPSC-derived CMs could serve as a platform for drug development and for design of personalized medication.

## Supporting Information

S1 Fig Confirmation of *exon 3 deletion*. ***Exon 3 deletion* of 05605.** CPVT cell line was also confirmed with PCR and agarose gel electrophoresis.
(TIF)

S2 Fig Ca^2+^ transient parameters as a response to dantrolene. (A) Diastolic Ca^2+^ level and (B) beating frequency in responder and non-responder CMs. Values during adrenaline perfusion were divided by values during dantrolene perfusion, separately for each cell. Green bars indicate responder CMs and purple bars non-responder CMs. Error bars, SEM. * indicates significant difference between adrenaline versus dantrolene within a group, *P<0.05. Numbers of cells analyzed in *exon 3 del* n = 13, *P2328S* n = 29, *T2538R* n = 11, *L4115F* n = 28, *Q4201R* n = 15, *V4653F* n = 10, Control (WT) n = 20.
(TIF)

S1 Protocol English translated trial study protocol. (DOCX)

S2 Protocol Trial study protocol in the original language (Finnish). (DOC)

S1 Table Primer sequences for EB RT-PCR. (DOCX)

S2 Table Differences between RyR2 mutations in their Ca^2+^ transient properties during baseline and adrenaline perfusion. CL indicates cluster numbers. Upward pointing arrow indicates significantly (p<0.05) higher and downward pointing arrow significantly lower diastolic Ca^2+^ level or beating frequency of the first mentioned mutation when compared to the second mentioned mutation. NS indicates that there was no statistical significance between mutations. As parallel pointing arrows between comparison groups indicate, the average of the beating frequency and diastolic Ca^2+^ level inside one mutation group corresponded and the average of these parameters decrease when moving from *P2328S* towards transmembrane area mutations.
(DOCX)
