# Correction: Antiarrhythmic Effects of Dantrolene in Patients with Catecholaminergic Polymorphic Ventricular Tachycardia and Replication of the Responses Using iPSC Models

**DOI:** 10.1371/journal.pone.0134746

**Published:** 2015-07-31

**Authors:** Kirsi Penttinen, Heikki Swan, Sari Vanninen, Jere Paavola, Annukka M. Lahtinen, Kimmo Kontula, Katriina Aalto-Setälä

There are errors in Fig 2, “Features of the PVCs,” and its caption. Please see the corrected Fig 2 and its caption here.

**Fig 2:**
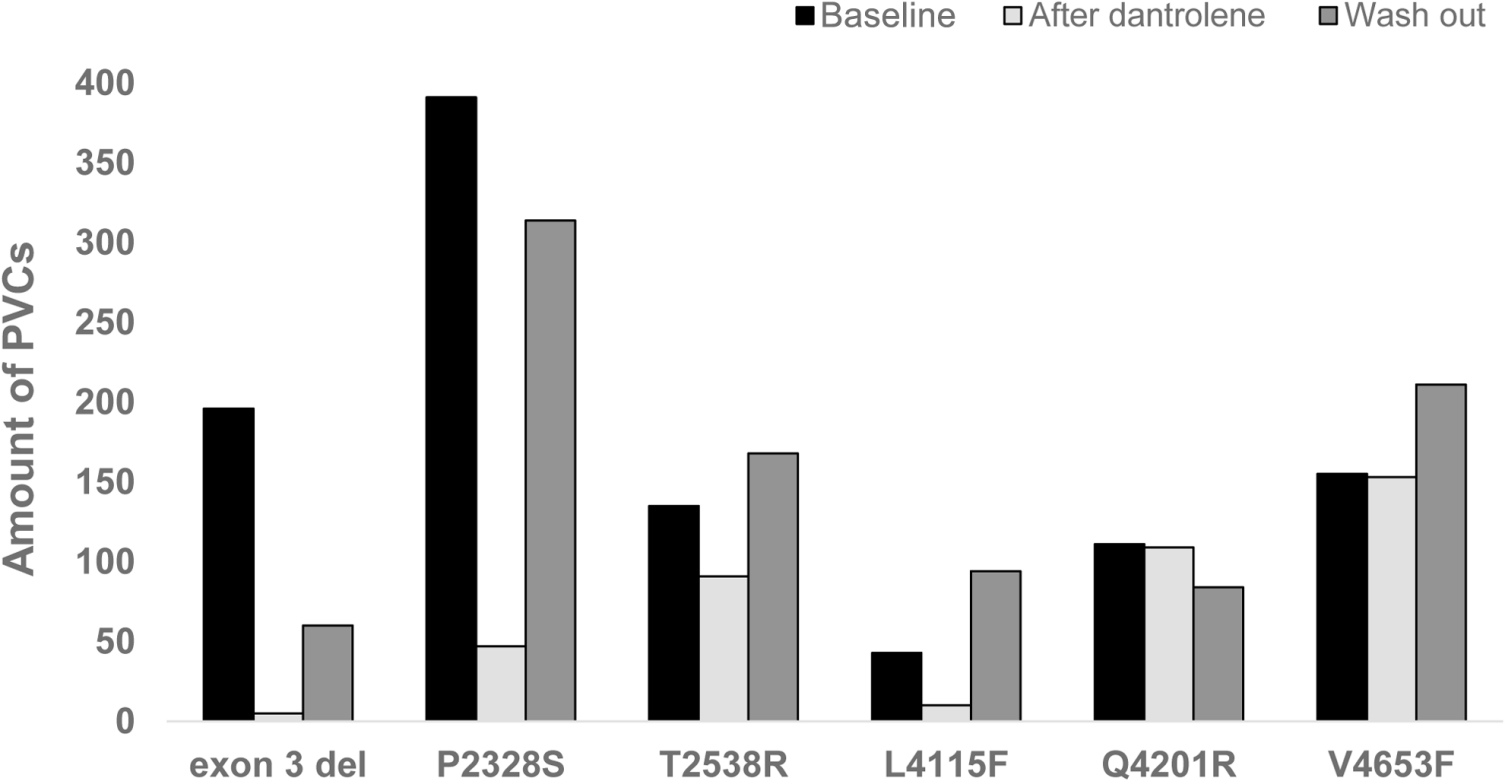
Features of the PVCs. Number of PVCs in exercise stress test before (baseline) and after administration of intravenous dantrolene and 24 hours after dantrolene wash out.

## References

[1] Penttinen K, Swan H, Vanninen S, Paavola J, Lahtinen AM, Kontula K, et al (2015) Antiarrhythmic Effects of Dantrolene in Patients with Catecholaminergic Polymorphic Ventricular Tachycardia and Replication of the Responses Using iPSC Models. PLoS ONE 10(5): e0125366 doi: 10.1371/journal.pone.0125366 25955245 10.1371/journal.pone.0125366 PMC4425399

