# Supplementary figures and images for: Antiarrhythmic Effects of Dantrolene in Patients with Catecholaminergic Polymorphic Ventricular Tachycardia and Replication of the Responses Using iPSC Models
Source: PLoS One. 2015 May 8;10(5):e0125366. doi: 10.1371/journal.pone.0125366 (PMC4425399; doi:10.1371/journal.pone.0125366)

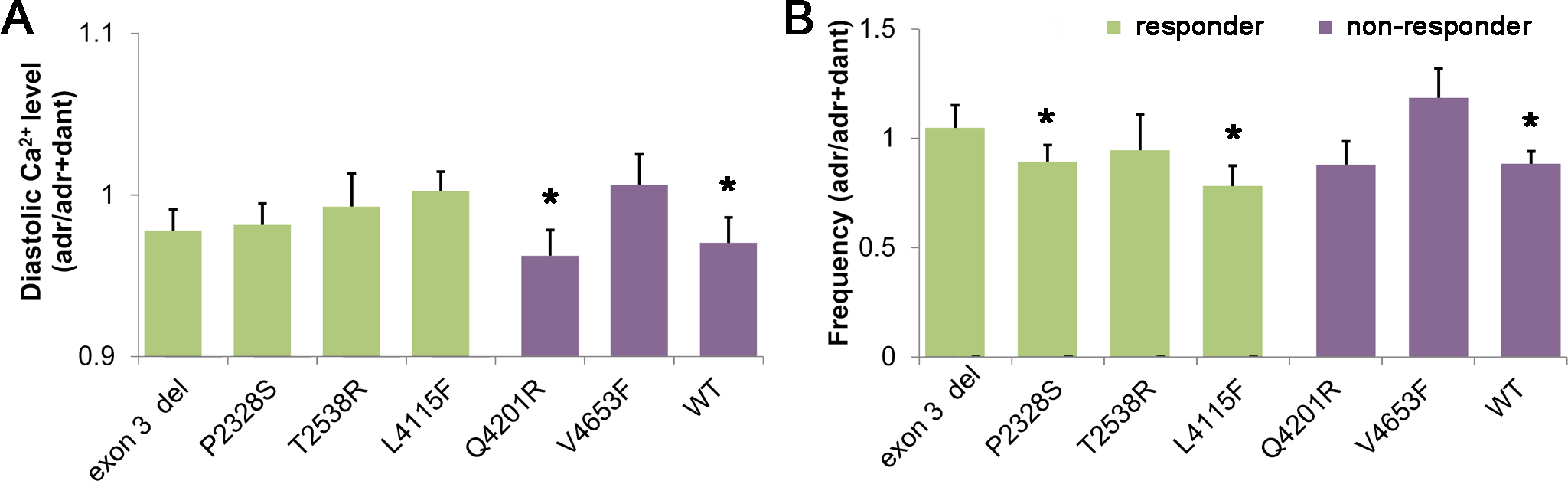

Supplement: S2 Fig — (A) Diastolic Ca2+ level and (B) beating frequency in responder and non-responder CMs. Values during adrenaline perfusion were divided by values during dantrolene perfusion, separately for each cell. Green bars indicate responder CMs and purple bars non-responder CMs. Error bars, SEM. * indicates significant difference between adrenaline versus dantrolene within a group, *P<0.05. Numbers of cells analyzed in exon 3 del n = 13, P2328S n = 29, T2538R n = 11, L4115F n = 28, Q4201R n = 15, V4653F n = 10, Control (WT) n = 20. (TIF) [file pone.0125366.s002.TIF]
