# Supplementary material for: Antiarrhythmic Effects of Dantrolene in Patients with Catecholaminergic Polymorphic Ventricular Tachycardia and Replication of the Responses Using iPSC Models
Source: PLoS One. 2015 May 8;10(5):e0125366. doi: 10.1371/journal.pone.0125366 (PMC4425399; doi:10.1371/journal.pone.0125366)
Supplement: S1 Protocol — (DOCX) [file pone.0125366.s003.docx]

**S1 Protocol.** English translated trial study protocol.

**Study protocol**

**Study title: Dantrolene in CPVT**

The purpose of the study is to study whether

1. dantrolene has an effect on ventricular arrhythmias induced during stress exercise test in patient carrying different mutations for CPVT
2. dantrolene has an effect on baseline beating rate, conduction or QTc in patient carrying different mutations for CPVT

The following parameters are monitored:

- sinus rhythm at baseline

- the amount of ventricular extrasystole at baseline during 5 minutes

- beating rate, where ventricular extrasystole start to appear during stress exercise test

- The amount of ventricular extrasystole /min during each ramp

- The longest ventricular tachycardia event during each ramp

- QTc interval is measured during baseline, during exercise test and during recovery

- the duration of the exercise test is measured (minutes)

- the beating rate (sinus rhythm) is measured at the end of each ramp

- the maximal beating rate is monitored (sinus rhythm)

**Patients to be recruited**

For this study 5-6 CPVT patients with a sequenced RyR2 mutation are recruited. Patients to be included have already earlier donated a skin biopsy for iPS cell studies. All the patients are on beta-blocker treatment, usually propranolol 80-160 mg/day or bisoprolol 5-10 mg/day. Other than CPVT, the patients are healthy. All the patients have already earlier participated studies about CPVT and for this study they are contacted either by phone or by a letter, where they will be informed about the study and the potential risks. All the patients will receive written information about the study as well as the informed consent form. The consent form will be signed during the first visit for the study. All participants will be on their regular medication during the entire study. This study does not affect the medication of the participants after the study. The participants do not benefit from this study at least in the near future, but the study aims at producing new data for future medication.

All the participants are Finnish speaking and they are all over 18 years of age, no pregnant nor lactating individuals are recruited. All the participants have performed earlier stress exercise tests and no problems or complications have been experienced with the previous tests. The following laboratory tests will be analyzed during the the first day before the first exercise test as well as on the morning of the second day: PVK, K, Na, fs-Ca, Krea. During dantrolene test, all the participants have an intra-venous (iv) infusion and thus a route for additional medication if needed during the tests in this proposal. The participants are covered by the insurance of the hospitals.

Exclusion criteria

- Calcium blocker treatment

- Pregnancy

- Any musculoskeletal problem affecting their performance

- Systolic blood pressure over 160 or diastolic over 95 mmHg

- Previously diagnosed coronary artery disease, congenital heart problem, or a pacemaker implanted for bradycardia

- ECG: PQ>200 ms, QRS>120 ms, RV5 or SV1>29 mm, delta-wave

**Study protocol**

**1. day**

All the participants take their beta-blocker medication normally in the morning before coming to the hospital. An iv-catheter is placed and saline is infused slowly during exercise test. The participants are in continuous ECG monitoring during all exercise test and during dantrolene infusion as well as 24 hours after the infusion. Blood pressure is monitored regularly with a cuff in the upper arm.

During the first exercise test, participants cycle 30 W + 15W/one minute ramps until about 80% of the maximum beating rate for the age. Due to the beta-blocker medication, it is possible that the desired heart is not reached.

The participants rest for at least 2 hours before the second exercise test. Before that dantrolene is infused 1.5 mg/kg. Dantrolene concentrate (20 mg) is diluted into 60 ml and infused in 5 minutes and thus the whole infusion time is about 30 minutes. The heart rate in continuously monitored for the next 24 hours as well as blood pressure is regularly analyzed.

1. **day**

The third exercise test is performed (about 18 hours after the second one).
