# Supplementary material for: Antiarrhythmic Effects of Dantrolene in Patients with Catecholaminergic Polymorphic Ventricular Tachycardia and Replication of the Responses Using iPSC Models
Source: PLoS One. 2015 May 8;10(5):e0125366. doi: 10.1371/journal.pone.0125366 (PMC4425399; doi:10.1371/journal.pone.0125366)
Supplement: S2 Protocol — (DOC) [file pone.0125366.s004.doc]

**S2 Protocol.** Trial study protocol in the original language (Finnish).

14.01.2013

**DANTROLEENI KATEKOLIAMIINIHERKÄSSÄ KAMMIOTAKYKARDIASSA**

**Tutkimus dantroleenin vaikutuksista perinnöllistä monimuotoista kammiotiheälyöntisyttä sairastavien potilaiden rytmihäiriöiden ilmaantuvuuteen sekä sydämen sähköiseen repolarisaatioon**

Koejärjestelyllä pyritään saamaan vastaus seuraaviin kysymyksiin:

- estääkö tai vähentääkö suonensisäisesti annettu kerta-annos dantroleenia kammioperäisten lisälyöntien ilmaantumista submaksimaalisessa rasituksessa perinnöllistä monimuotoista kammiotiheälyöntisyyttä sairastavilla verrattuna ennen lääkkeen antoa vallinneeseen tilaan (beetasalpaajalääkityksen käytössä ollessa)

- vaikuttaako dantroleeni leposykkeeseen, johtumiseen tai QTc-aikaan perinnöllistä monimuotoista kammiotiheälyöntisyyttä sairastavilla

Tutkimuksessa mitataan seuraavia tekijöitä:

- sinustaajuus levossa

- kammiolisälyöntien määrä levossa 5 minuutin aikana

-syketaso, jossa kammiolisälyönnit ilmaantuvat ensi kertaa rasituksen aikana

-kammiolisälyöntien määrä/minuutti kunkin rasitusportaan aikana

-pisimmän perättäisten kammiokompleksien sarjan pituus kunkin rasitusportaan (á 1 min) aikana

-sydämen sähköisen palautumisen muutoksia mitataan lepo-EKG:sta sekä rasituksen ja sitä seuraavan palautumisen aikana rekisteröidystä EKG:sta QT -ajan pituutta seuraamalla.

- rasituksen kesto minuuteissa

- sinustaajuus kunkin kuormaportaan päättyessä

- saavutettu maksimaalinen sinustaajuus

**Tutkittavat potilaat**

Pilottiluonteiseen tutkimukseen otetaan mukaan 5-6 genotyypiltään tunnettua RYR2 -potilasta, joilla on kliinisesti todettu RYR2 -mutaatiosta johtuva perinnöllinen monimuotoinen kammiotiheälyöntisyys. Ensisijaisesti tutkimukseen kutsutaan potilaita, joista on jo iPS-soluviljelmät *in vitro*-tutkimuksia varten. Tutkimukseen otettavilla on jo käytössään beetasalpaajalääkitys, tavallisesti propranololia 80-160 mg/vrk tai bisoprololia 5-10 mg/vrk ja he ovat perinnöllistä monimuotoista kammiotiheälyöntisyyttä lukuunottamatta muuten terveitä. Tutkittavia, jotka ovat olleet jo tutkimusryhmän muissa tutkimuksissa aiemmin mukana, pyydetään tutkimukseen puhelimitse, jolloin heille selostetaan tutkimuksen kulku ja mahdolliset riskit suullisesti ja heidän alustavan suostumuksensa jälkeen heille toimitetaan kirjallinen informaatio tutkimuksesta postitse. Tutkittavat allekirjoittavat suostumuslomakkeensa tutkimukseen saapuessaan. Tutkittavien hoito jatkuu tutkimuksen ajan ja sen jälkeen ennallaan. Tutkimus ei vaikuta tutkittavien potilaiden hoitoon välittömästi mutta saattaa mahdollistaa täydentävän hoidon käyttöönoton olemassa olevan lisäksi lisätutkimusten jälkeen tutkittaville tai heidän perheenjäsenilleen.

Tutkittavat ovat kaikki suomenkielisiä. Tutkimus ei kohdistu vajaakykyisiin, alaikäisiin, ras-kaana oleviin eikä imettäviin naisiin, eikä vankeihin. Tutkittavissa ei ole muita myöskään muita hen-kilöryhmiä, joiden vapaaehtoisuus voidaan kyseenalaistaa. Tutkittaville on tehty rasituskokeita toistu-vasti aiemmin eikä niiden suorittamiseen ole liittynyt ongelmia tai komplikaatioita. Rasituskokeen yh-teydessä on tavanomaisen komplikaatioiden hoitovalmiuden lisäksi myös nopea mahdollisuus suonen-sisäiseen nesteen- ja lääkkeenantoon tutkimuksen alussa asetettavan laskimokanyylin vuoksi. Tutkit-tavien vakuutusturva sisältyy sairaalalle projektipotilaista maksettaviin käynti- ja toimenpidemaksuihin.

Poissulkukriteereinä on

- kalsiumkanavasalpaaja- tai metoklopramidilääkitys

- raskaus

- liikuntaa rajoittava tuki- ja liikuntaelimistön sairaus

- tarve kuljettaa moottoriajoneuvoa lääkkeen antoa seuraavan vuorokauden aikana

- verenpaine levossa yli 160 (S) tai yli 95 (D)

- aiemmin todettu sepelvaltimotauti, läppävika, synnynnäinen sydänvika, tahdistinhoito

- EKG: PQ>200 ms, QRS>120 ms, RV5 tai SV1>29 mm, delta-aalto

**TUTKIMUKSEN KULKU**

**1. päivä**

Tutkittavat ottavat tutkimukseen tullessaan ja tutkimuksen aikana beetasalpaajalääkityksensä tavanomaiseen tapaan. Tutkittavalle asetetaan laskimokanyyli, jonka kautta infusoidaan hitaasti fysiologista keittosuolaa kunkin rasituskokeen aikana. Potilailla on lääkkeiden annon, rasituskokeiden ja niiden jälkeisen palautumisvaiheen ajan jatkuva EKG -seuranta sekä olkavarsimansetilla suoritettava verenpaineseuranta.

Suoritetaan ensimmäinen rasituskoe polkupyöräergometrilla, jossa potilas polkee 30 W + 15 W/1 min portain syketasolle, joka on n. 80 % iänmukaisesta maksimista. Koska potilailla on jo käytössään beetasalpaajalääkitys, saattaa saavutettava syketaso jäädä kuitenkin tätä matalammaksi.

Vähintään 2 tunnin lepotauon jälkeen suoritetaan toinen rasituskoe, jota ennen infusoidaan dantroleenia 1.5 mg/kg. Kukin 20 mg:n infuusiokonsentraattipakkaus laimennetaan 60 ml:n nestemäärään, joka infusoidaan n. 5 minuutissa, jolloin painon mukainen kokonaislääkeannoksen infuusion kesto on n. 30 minuuttia. Tutkittavia seurataan sairaalassa sydämen rytmiä monitoroiden seuraavaan aamuun asti.

1. **päivä**

Suoritetaan edellä kerrotulla tavalla kolmas rasituskoe (n. 18 tuntia edellisestä).

**Tutkimuskeskukset**

Tutkimus suoritetaan HYKSin Kardiologian klinikassa Sydäntutkimusosastolla tai Kolmiosairaalan vuodeosastolla (3-4 potilasta) sekä TAYSissä (2 potilasta).

**POTILAIDEN JA VERROKKIHENKILÖIDEN SUOSTUMUS**

Potilaille ja verrokkihenkilöille kerrotaan tutkimuksen tarkoitus, sen kulku ja mahdolliset haitat suullisesti ja tiedotteella, joka on tämän tutkimussuunnitelman liitteenä. Tutkimuksiin osallistuville ei makseta palkkiota.

## TIEDOTE TUTKIMUKSESTA

**DANTROLEENI KATEKOLIAMIINIHERKÄSSÄ KAMMIOTAKYKARDIASSA**

Sinua pyydetään mukaan tutkimukseen, jonka tarkoituksena on selvittää, vähentääkö sydänlihassolujen kalsiumkanaviin vaikuttava dantroleenilääke rasituksessa ilmeneviä rytmihäiriöitä rytmihäiriösairaudessasi. Olemme arvioineet, että soveltuisit mukaan tutkimukseen, koska sairastat perinnöllistä katekoliamiiniherkkää kammiotakykardiaa (CPVT). Tämä tiedote kuvaa tutkimusta ja osuuttasi siinä.

## Osallistumisen vapaaehtoisuus

Osallistuminen tähän tutkimukseen on täysin vapaaehtoista. Voit kieltäytyä osallistumasta tutkimukseen tai keskeyttää osallistumisesi syytä ilmoittamatta milloin tahansa. Sinun ei tarvitse osallistua tähän tutkimukseen saadaksesi hoitoa. Lääkärisi kertoo Sinulle sairautesi hoitovaihtoehdoista. Tutkijalääkäri voi joutua keskeyttämään osallistumisesi. Jos näin tapahtuu, kanssasi keskustellaan lopettamiseen liittyvistä jatkotoimenpiteistä.

Lue rauhassa tämä tiedote. Jos Sinulla on kysyttävää, voi olla yhteydessä tutkijalääkäriin tai tutkimushoitajana. Jos päätät sallistua tutkimukseen, pyydämme Sinua allekirjoittamaan liitteenä olevan suostumuslomakkeen.

## Tutkimuksen toteuttaja

Tämän tutkimuksen toteuttavat HYKSin Meilahden sairaalassa dos. Heikki Swan ja TAYSin Sydänkeskuksessa dos Katriina Aalto-Setälä. Tutkimuksen rekisterinpitäjä on dos Heikki Swan tutkimusryhmineen, joka vastaa tutkimuksen yhteydessä tapahtuvan henkilötietojen käsittelyn lainmukaisuudesta.

## Tutkimuksen tausta ja tarkoitus

Perinnöllinen polymorfinen kammiotakykardia on harvinainen, pahanlaatuinen rytmihäiriösairaus. Kyseessä on sydämen sähköisen toiminnan häiriö. Rakenteellista sydänvikaa ei ole todettavissa ja levossa tutkittu EKG:kin (sydänfilmi) on tautia kantavilla normaali. Potilaiden rytmihäiriöt ilmenevät tyypillisesti aina syketason noustua tietyn yksilöllisen kynnyksen yläpuolelle. Taudin toteamisessa keskeinen menetelmä onkin rasituskoe, jossa alun normaalin rytmin kiihtyessä rasituksen myötä alkaa ilmaantua kammiolisälyöntisyyttä yhä enenevässä määrin. Rytmihäiriöalttius johtuu sydänlihassolujen sisäisestä kalsiumaineenvaihdunnan häiriöstä.

Oireyhtymää sairastavien potilaiden hoidossa käytetään tavallisesti beetasalpaajalääkitystä, josta on parhaat kokemukset rytmihäiriöiden estossa. Beetasalpaajat ovat lääkkeitä, joita käytetään myös mm. sepelvaltimotautia ja verenpainetautia sairastavien hoidossa. Beetasalpaajat eivät yleensä poista rytmihäiriöiden ilmaantumista rasituksessa mutta kokemukset viittaavat siihen, että alttius pahemmanlaatuisiin rytmihäiriöihin, joiden seurauksena verenkierto saattaisi romahtaa, pienenee. Beetasalpaajalääkityksen aikana on harvoin esiintynyt tajunnanmenetyskohtauksia. Eräät beetasalpaajalääkkeet ovat vasta-aiheisia mm. astmaa sairastavilla. Vain harvoin beetasalpaajalääkitystä ei voida käyttää sen sivuvaikutusten vuoksi.

Tarkoituksenamme on selvittää, vähentääkö sydänlihassolujen kalsiumkanaviin vaikuttava dantroleenilääke rasituksessa ilmeneviä rytmihäiriöitä rytmihäiriösairaudessasi. Mikäli rytmihäiriöiden ilmaantuminen tutkimuslääkkeen antamisen jälkeen olisi vähäisempää kuin beetasalpaajalääkityksen käytössä ollessa, voitaisiin kyseistä lääkettä edelleen tutkimalla mahdollisesti löytää lisälääke niille perinnöllistä monimuotoista kammiotiheälyöntisyyttä sairastaville potilaille, joille beetasalpaajalääkitys ei yksinään ole joko riittävä tai jotka eivät sitä sivuvaikutusten vuoksi voi käyttää.

Tutkimukseen pyydetään mukaan henkilöitä, jotka ovat täysi-ikäisiä ja joilla on RYR2-geenin mutaatiosta johtuva perinnöllinen katekoliamiiniherkkä kammiotiheälyöntisyys ja sen hoidoksi käytössään beetasalpaajalääkitys mutta ei muita rytmihäiriölääkkeitä eikä muita merkittäviä sydän- ja verisuonisairauksia. Tutkimukseen osallistuu noin 5-6 tutkittavaa.

## Tutkimusmenetelmät ja tutkimuksen toimenpiteet

Tutkimukseen osallistuminen kestää runsaan vuorokauden, jonka ajan olet sairaalassa. Ensimmäisenä tutkimuspäivänä otetaan verinäyte ja asetetaan laskimoon muovikanyyli (”tippa”) tutkimuslääkkeiden antoa varten. Ensimmäisenä päivänä suoritat rasituskokeen kahdesti. Ennen toista rasituskoetta, annetaan laskimoon dantroleenilääkettä. Kolmas rasitus-koe tehdään seuraavana päivänä, minkä jälkeen voit kotiutua. Lääkkeen vaikutusta verenpai-neeseen ja sykkeeseen seurataan tutkimuksen aikana EKG:n ja verenpainemittausten avulla.

Käyttämistäsi lääkkeistä tutkimuksen alkaessa sinun tulee ilmoittaa tutkijoille. Tutkimuksen aikana mahdollisesti potilaan tai koehenkilön tarvitsemien muiden lääkkeiden sekä tupakan tai alkoholin käyttöön on pyydettävä tutkijoiden lupa.Tutkimuksen aikana kaikkien hedelmällisessä iässä olevien naisten on käytettävä luotettavaa ehkäisyä. Tutkijalääkäri keskustelee tarvittaessa kanssasi käyttämästäsi ehkäisymenetelmästä. Raskaana olevat, imettävät tai raskautta suunnittelevat naiset eivät voi osallistua tähän tutkimukseen.

## Tutkimuksen mahdolliset hyödyt

Dantroleenilääkkeen tutkiminen ei vaikuta välittömästi hoitoosi mutta tutkimus voi auttaa uuden lääkehoidon löytymisessä rytmihäiriöiden ehkäisemiseksi. Tutkittavasta sairaudesta voidaan myös saada hyödyllistä lisätietoa. Saat tietoa terveydentilastasi tutkimuksen aikana tehtävistä lääkärintarkastuksista ja laboratoriokokeista.

## Tutkimuksesta mahdollisesti aiheutuvat haitat ja epämukavuudet

Tutkimuksessa tarvittavan laskimokanyylin asettaminen on tavallinen toimenpide sydänpotilaiden tutkimuksessa ja hoidossa mutta joskus toimenpiteeseen saattaa liittyä paikallista kipua tai verenpurkauman muodostumista ihonalaiskudokseen. Lääkkeen sivuvaikutuksena saattaa esiintyä lyhytaikaista lihasvoiman heikentymistä tai paikallista laskimoärsytystä lääkkeenantokohdassa. Rasituskokeeseen liittyvä rytmihäiriöriski on samankaltainen kuin muussakin samalla tavalla kuormittavassa liikunnassa. Mahdollisiin rytmihäiriöongelmiin ja verenpaineen laskuun on varauduttu kuten rasituskoetta suoritettaessa yleensäkin varaudutaan.Tutkijalääkäri voi kertoa Sinulle muista mahdollisista haitoista.

Tutkimuslääkkeen käyttöön saattaa liittyä ennalta tuntemattomia riskejä. Jos tutkimuksen aikana saadaan turvallisuutesi kannalta oleellista uutta tietoa tutkimusvalmisteesta, tutkijalääkäri ottaa Sinuun välittömästi yhteyttä ja keskustelee kanssasi siitä, haluatko edelleen jatkaa tutkimuksessa.

## Tietojen luottamuksellisuus ja tietosuoja

Tutkimuksessa henkilöllisyytesi sekä muut tunnistettavat tiedot ovat ainoastaan tutkimuksen henkilökunnan tiedossa, ja he kaikki ovat salassapitovelvollisia. Tutkimukseen liittyvistä tutkimustuloksista, selvityksistä tai julkaisuista ei yksittäisiä tutkittavia voi tunnistaa.

Tutkimusrekisteristä on laadittu henkilötietolain 10 §:n mukainen rekisteriseloste, jonka saat halutessasi nähtäväksi.

Terveydentilaasi koskevia ja tutkimuksen kannalta tarpeellisia tietoja voidaan luvallasi kerätä myös muista terveydenhuollon toimintayksiköistä. Tutkijalääkäri voi tällöin hankkia tarvitsemansa tiedot henkilötunnuksesi avulla. Sinulla on oikeus tarkastaa omat henkilötietosi ja tarvittaessa pyytää niihin korjauksia.

Suomessa lääkevalvontaviranomaisella (Lääkealan turvallisuus- ja kehittämiskeskus Fimea) on oikeus varmistaa, että tutkimustiedot on hankittu ja tutkimus toteutettu asianmukaisella tavalla. Kaikissa tapauksissa tietojasi käsitellään luottamuksellisesti.

Jos osallistumisesi tutkimukseen jostain syystä keskeytyy, keskeyttämiseen mennessä kerättyjä tietoja käytetään osana tutkimusaineistoa ja lääkkeen turvallisuusarviointia.

## Tutkimuksen kustannukset ja taloudelliset selvitykset

Tutkimus on Sinulle maksuton. Tutkimuskäynneistä aiheutuvat mahdolliset matkakustannukset korvataan todellisten kustannusten mukaan tositteiden perusteella.

Tutkimus rahoitetaan sairaalan tutkimusmäärärahoista ja Suomen Sydäntutkimussäätiön apurahalla.

## Tutkittavien vakuutusturva

Jos tutkimuslääkkeestä tai tutkimuksen takia tehdystä toimenpiteestä aiheutuu Sinulle henkilövahinko, voit hakea korvausta potilasvakuutuksesta. Se korvaa potilasvahinkolain mukaisesti terveyden ja sairaanhoidon yhteydessä aiheutuneita henkilövahinkoja laissa tarkemmin säädellyin edellytyksin. Potilasvakuutuskeskus huolehtii potilasvahinkojen korvauskäsittelystä.

## Tutkimuksen päättyminen

Hoitosi jatkuu nykyisellään tutkimuksen jälkeen. Tarvittaessa tutkijalääkäri keskustelee kanssasi hoidostasi tutkimuksen päättymisen jälkeen.

## Lisätietoja

Jos Sinulla on kysyttävää tutkimuksesta, voit olla yhteydessä tutkijalääkäriin tai muuhun tutkimuksen henkilökuntaan. Heidän kanssaan voit keskustella kaikista tutkimuksen aikana mahdollisesti ilmenneistä haittavaikutuksista, epäilyttävistä oireista ja muista mieltäsi askarruttavista asioista.

Tutkimukseen liittyvissä asioissa yhdyshenkilönä on Meilahden sairaalassa dos. Heikki Swan, HYKS Sydäntutkimusosasto, Haartmanink. 4, 00290 Helsinki, puhelin 050-4286591 tai 09-4711, Tampereen Yliopistosairaalassa dos. Katriina Aalto-Setälä, TAYS Sydänkeskus, Biokatu 6, 33520 Tampere, puhelin03 - 311 66088 tai 040-5829567.

# SUOSTUMUS LÄÄKETUTKIMUKSEEN

Minua on pyydetty osallistumaan dantroleeni-lääkkeellä suoritettavaan rytmihäiriöiden hoitotutkimukseen.

Olen saanut, lukenut ja ymmärtänyt tutkimuksesta kertovan tiedotteen 23.11.2012. Tiedotteesta olen saanut riittävän selvityksen tutkimuksesta (dantroleeni katekoliamiiniherkässä kammiotakykardiassa) ja sen yhteydessä suoritettavasta tietojen keräämisestä, käsittelystä ja luovuttamisesta. Tiedotteen sisältö on kerrottu minulle myös suullisesti ja olen saanut riittävän vastauksen kaikkiin tutkimusta koskeviin kysymyksiini. Tiedot antoi ________________________________ , ___/___/ 20__ . Minulla on ollut riittävästi aikaa harkita osallistumistani tutkimukseen.

Minulle kerrotaan, mistä minua koskevia tietoja hankitaan. Annan luvan itseäni koskevien, tutkimuksen kannalta tarpeellisten tietojen keräämiseen perinnöllisten rytmihäiriösairauksien tutkimusrekisteriin. Tietoja voidaan lääketutkimuksen sitä edellyttäessä pyytää niistä terveydenhoitopaikoista, joissa on potilastietojani. Tätä tarkoitusta varten lääkäri saa kirjata henkilötunnukseni sekä käyttää sitä tietojen saamiseksi.

Kaikki minusta tutkimuksen aikana kerättävät tiedot käsitellään luottamuksellisina.

Lääkevalvonnasta ja –turvallisuudesta vastaavan viranomaisen, Suomessa Lääkealan turvallisuus- ja kehittämiskeskus Fimean edustajilla on oikeus varmistaa tutkimustietojen totuudenmukaisuus ja tutkimuksen asianmukainen suorittaminen. Tämä tapahtuu vertaamalla tutkimustietoja alkuperäisiin sairaskertomuksiini ja terveydentilaani koskeviin tietoihin. edellä mainitut tahot ovat velvollisia pitämään tiedot salassa.

Tässä tutkimuksessa kerättäviä tietoja voidaan käsitellä muualla kuin tiedot keränneen tutkijalääkärin tiloissa ja laitteissa. Tällöin tiedot ovat koodatussa muodossaan.

Tässä lääketutkimuksessa kerättävä tieto voi olla hyödyksi myös selvitettäessä dantroleeni-lääkkeen uutta käyttötarkoitusta, josta tämän suostumuksen antamisen hetkellä ei ole tietoa. Hyväksyn, että minusta kerättäviä tietoja voidaan viranomaisen luvalla käyttää tällaiseen tarkoitukseen.

Ymmärrän, että osallistumiseni tähän tutkimukseen on täysin vapaaehtoista. Minulla on oikeus milloin tahansa tutkimuksen aikana ja syytä ilmoittamatta keskeyttää tutkimukseen osallistuminen. Tutkimuksesta kieltäytyminen tai sen keskeyttäminen ei vaikuta jatkohoitooni. Olen tietoinen siitä, että minusta keskeyttämiseen mennessä kerättyjä tietoja käytetään osana tutkimusaineistoa ja lääkkeen turvallisuusarviointia.

Allekirjoituksellani vahvistan osallistumiseni tähän tutkimukseen ja suostun vapaaehtoisesti tutkimushenkilöksi.

________________________ _________________________

Allekirjoitus Päiväys

_________________________ _________________________

Nimen selvennys Syntymäaika

__________________________________________________________

Osoite

Suostumus vastaanotettu

_________________________ __________________________

Tutkijalääkärin allekirjoitus Päiväys

_________________________

Nimen selvennys

# Alkuperäinen allekirjoitettu tutkimushenkilön suostumus sekä kopio tutkimushenkilötiedotteesta jäävät tutkijalääkärin arkistoon. Tutkimushenkilötiedote ja kopio allekirjoitetusta suostumuksesta annetaan tutkimushenkilölle.

**Kirjallisuus**

[Kim JY](http://www.ncbi.nlm.nih.gov/pubmed?term=Kim JY%5BAuthor%5D&cauthor=true&cauthor_uid=21878205), [Chun S](http://www.ncbi.nlm.nih.gov/pubmed?term=Chun S%5BAuthor%5D&cauthor=true&cauthor_uid=21878205), [Bang MS](http://www.ncbi.nlm.nih.gov/pubmed?term=Bang MS%5BAuthor%5D&cauthor=true&cauthor_uid=21878205), [Shin HI](http://www.ncbi.nlm.nih.gov/pubmed?term=Shin HI%5BAuthor%5D&cauthor=true&cauthor_uid=21878205), [Lee SU](http://www.ncbi.nlm.nih.gov/pubmed?term=Lee SU%5BAuthor%5D&cauthor=true&cauthor_uid=21878205). Safety of low-dose oral dantrolene sodium on hepatic function. [Arch Phys Med Rehabil.](http://www.ncbi.nlm.nih.gov/pubmed/21878205#%23) 2011 Sep;92:1359-63.

Flewellen EH, Nelson TE, Jones WP, Arens JF, Wagner DL. Dantrolene dose response in awake man: implications for management of malignant hyperthermia. Anesthesiology 1983; 59: 275-80.

Brandom BW, Larach MG, Chen MSA, Young MC. Complications associated with the administration of dantrolene 1987 to 2006: a report from the North American malignant hyperthermia registry of the malignant hyperthermia association of the United States. Anesth Analg 2011; 112: 1115-1123.
