# Supplementary material for: Antiarrhythmic Effects of Dantrolene in Patients with Catecholaminergic Polymorphic Ventricular Tachycardia and Replication of the Responses Using iPSC Models
Source: PLoS One. 2015 May 8;10(5):e0125366. doi: 10.1371/journal.pone.0125366 (PMC4425399; doi:10.1371/journal.pone.0125366)
Supplement: S1 Table — (DOCX) [file pone.0125366.s005.docx]

# **S1 Table. Primer sequences for EB RT-PCR.**

| **Gene** | **Forward Primer** | **Reverse Primer** |
| --- | --- | --- |
| **Endodermal** |  |  |
| *AFP* | AGAACCTGTCACAAGCTGTG | GACAGCAAGCTGAGGATGTC |
| *SOX-17* | CGCACGGAATTTGAACAGTA | CACACGTCAGGATAGTTGCAG |
| **Ectodermal** |  |  |
| *Nestin* | CAGCTGGCGCACCTCAAGATG | AGGGAAGTTGGGCTCAGGACTGG |
| *SOX-1* | AAAGTCAAAACGAGGCGAGA | AAGTGCTTGGACCTGCCTTA |
| **Mesodermal** |  |  |
| *VEGF-R2* | GGAGTTATGGTGGGTATGGGTC | AGTGGTGACAAAGGAGTAGCCA |
