# Supplementary material for: Antiarrhythmic Effects of Dantrolene in Patients with Catecholaminergic Polymorphic Ventricular Tachycardia and Replication of the Responses Using iPSC Models
Source: PLoS One. 2015 May 8;10(5):e0125366. doi: 10.1371/journal.pone.0125366 (PMC4425399; doi:10.1371/journal.pone.0125366)
Supplement: S2 Table — CL indicates cluster numbers. Upward pointing arrow indicates significantly (p<0.05) higher and downward pointing arrow significantly lower diastolic Ca2+ level or beating frequency of the first mentioned mutation when compared to the second mentioned mutation. NS indicates that there was no statistical significance between mutations. As parallel pointing arrows between comparison groups indicate, the average of the beating frequency and diastolic Ca2+ level inside one mutation group corresponded and the average of these parameters decrease when moving from P2328S towards transmembrane area mutations. (DOCX) [file pone.0125366.s006.docx]

|  | **CLUSTERS** | **MUTATIONS** | **BASELINE** | | **ADRENALINE** | |
| --- | --- | --- | --- | --- | --- | --- |
|  |  |  | **Diastolic Ca^2+^  level** | **Beating frequency** | **Diastolic Ca^2+^  level** | **Beating frequency** |
| **Exon 3 deletion**  **versus**  **point mutations** | CL 1 vs. CL 2 | exon 3 del vs. P2328S | ***↓*** | ***↓*** | ***↓*** | ***↓*** |
|  | CL 1 vs. CL2-3 | exon 3 del vs. T2538R | ***↓*** | ***↓*** | ***↓*** | ***↓*** |
|  | CL 1 vs. CL 3 | exon 3 del vs. L4115F | ***↓*** | ***↓*** | ***↓*** | ***↓*** |
|  | CL 1 vs. CL3 | exon 3 del vs. Q4201R | ***↓*** | ***↓*** | ***↓*** | ***↓*** |
|  | CL 1 vs. CL 4 | exon 3 del vs. V4653F | NS | ***↓*** | NS | ***↓*** |
| **Comparison of point mutations**  **exceeding towards transmembrane area** | CL 2 vs. CL 2-3 | P2328S vs. T2538R | NS | NS | ***↑*** | ***↑*** |
|  | CL 2 vs. CL 3 | P2328S vs. L4115F | ***↑*** | ***↑*** | ***↑*** | ***↑*** |
|  | CL 2 vs. CL 3 | P2328S vs. Q4201R | ***↑*** | ***↑*** | ***↑*** | ***↑*** |
|  | CL 2 vs. CL 4 | P2328S vs. V4653F | ***↑*** | ***↑*** | ***↑*** | ***↑*** |
|  | CL 2-3 vs. CL 3 | T2538R vs. L4115F | ***↑*** | NS | ***↑*** | NS |
|  | CL 2-3 vs. CL 3 | T2538R vs. Q4201R | ***↑*** | ***↑*** | ***↑*** | NS |
|  | CL 2-3 vs. CL 4 | T2538R vs. V4653F | ***↑*** | NS | ***↑*** | NS |
|  | CL 3 vs. CL 3 | L4115F vs. Q4201R | NS | NS | NS | NS |
|  | CL 3 vs. CL 4 | L4115F vs. V4653F | ***↑*** | NS | ***↑*** | NS |
|  | CL 3 vs. CL 4 | Q4201R vs. V4653F | ***↑*** | NS | ***↑*** | NS |

**S2 Table.** **Differences between RyR2 mutations in their Ca^2+^ transient properties during baseline and adrenaline perfusion.** CL indicates cluster numbers. Upward pointing arrow indicates significantly (p<0.05) higher and downward pointing arrow significantly lower diastolic Ca^2+^ level or beating frequency of the first mentioned mutation when compared to the second mentioned mutation. NS indicates that there was no statistical significance between mutations. As parallel pointing arrows between comparison groups indicate, the average of the beating frequency and diastolic Ca^2+^ level inside one mutation group corresponded and the average of these parameters decrease when moving from P2328S towards transmembrane area mutations.
